# Draft Genome Sequences of *Salmonella enterica* subsp. *enterica* Serovars Typhimurium and Nottingham Isolated from Food Products

**DOI:** 10.1128/genomeA.00699-16

**Published:** 2016-07-21

**Authors:** Hua Wang, Jie Zheng, Sherry Ayers, David C. Melka, Phillip E. Curry, Justin S. Payne, Anna Laasri, Charles Wang, Thomas S. Hammack, Eric W. Brown

**Affiliations:** a Division of Microbiology, Center for Food Safety and Applied Nutrition, Food and Drug Administration, College Park, Maryland, USA; b Division of Animal and Food Microbiology, Center for Veterinary Medicine, Food and Drug Administration, Laurel, Maryland, USA

## Abstract

A quantitative real-time PCR (qPCR) designed to detect *Salmonella enterica* subsp. *enterica* serovar Enteritidis, targeting the *sdf* gene, generated positive results for *S. enterica* subsp. *enterica* serovar Typhimurium (CFSAN033950) and *S. enterica* subsp. *enterica* serovar Nottingham (CFSAN006803) isolated from food samples. Both strains show pulsed-field gel electrophoresis (PFGE) patterns distinct from those of *S*. Enteritidis. Here, we report the genome sequences of these two strains.

## GENOME ANNOUNCEMENT

*Salmonella enterica* subsp. *enterica* serovar Enteritidis has become one of the primary causes of salmonellosis worldwide, accounting for 36% of the 403 outbreaks in 1998 to 2008 in the United States (1). Traditionally, the major sources of human *S*. Enteritidis infections are contaminated eggs and poultry; however, *S*. Enteritidis outbreaks have now been associated with a wide variety of other foods (1). A quantitative real-time PCR (qPCR) assay has been designed, targeting the *Salmonella* difference fragments (Sdf) to be able to screen *S*. Enteritidis from environmental and food samples, because the *sdf* gene had been reported only in *S*. Enteritidis strains from a wide range of clinical and environmental samples (2). However, a *S. enterica* subsp. *enterica* serovar Typhimurium strain (CFSAN033950) isolated from an imported Madras curry powder product and a strain of *S. enterica* subsp. *enterica* serovar Nottingham (CFSAN006803) isolated from a frog leg were tested as Sdf positive by *S*. Enteritidis qPCR.

Both strains were isolated using the Bacteriological Analytical Manual (BAM) culture method (http://www.fda.gov/Food/FoodScienceResearch/LaboratoryMethods/ucm070149.htm) and then serotyped with traditional serological methods (3) and the Luminex *Salmonella* serotyping assay (4–6). Pulsed-field gel electrophoresis (PFGE) performed using restriction enzyme XbaI exhibits unique PFGE patterns from both strains (http://www.cdc.gov/pulsenet/PDF/ecoli-shigella-salmonella-pfge-protocol-508c.pdf).

The genomic DNA from each strain was isolated from overnight cultures using the DNeasy blood and tissue kit (Qiagen, Valencia, CA), and their genomes were sequenced using an Illumina MiSeq (Illumina, San Diego, CA), with libraries prepared using a Nextera XT kit (Illumina), according to the manufacturer’s instructions. Genomic sequence contigs were assembled using SPAdes software version 3.6.2, and the sequence was annotated using the NCBI Prokaryotic Genome Automatic Annotation Pipeline (7). Genomic comparison was performed using CLC Genomics Workbench 8.5.1 (CLC bio, Waltham, MA).

The draft genome for *S*. Typhimurium (CFSAN033950) is 4,875,479 bp long and contains the Sdf regions V, IX, and the first 261 bp of region VII, which have been reported only in *S*. Enteritidis CAHFS-5 (2). Strain CFSAN033950 also carries all 6 genes of *Salmonella* difference region I (Sdr I): *lygA*, *lygB*, *lygC*, *lygD*, *lygE*, and *lygF*, which have been reported as unique to *S*. Enteritidis CAHFS-285 (2). However, these genes are arranged differently from their arrangement in *S*. Enteritidis CAHFS-285 and dispersed within a 7-kb region in the same contig. The draft genome for *S*. Nottingham (CFSAN006803) is 4,636,370 bp long and contains the Sdf regions III, IV, and the first 261 bp of region VII. This strain also carries the same 6 genes of Sdr I and displays the same gene pattern as CFSAN033950.

In retrospect, as both strains carry complete *lygD* genes, which are the targets of the *S*. Enteritidis qPCR, it is not surprising that these strains should test positive by *S*. Enteritidis qPCR. Additional investigation of the phylogeny of this Sdr I region in *Salmonella* may provide important insights for improving molecular serotyping of *Salmonella* species.

### Nucleotide sequence accession numbers.

The whole-genome shotgun projects for strains *S*. Typhimurium CFSAN033950 and *S*. Nottingham CFSAN006803 have been deposited in DDBJ/EMBL/GenBank under accession numbers LXNM00000000 and LXIQ00000000, respectively. The versions described in this paper are the first versions, LXNM01000000 and LXIQ01000000.
